# Supplementary material for: Conditional chemoconnectomics (cCCTomics) as a strategy for efficient and conditional targeting of chemical transmission
Source: eLife. 2024 Apr 30;12:RP91927. doi: 10.7554/eLife.91927 (PMC11060718; doi:10.7554/eLife.91927)
Supplement: Figure 3—source data 3. [file elife-91927-fig3-data3.zip › Figure 3 source data3 part 2.zip!/Figure 3-Source data 3-part2/1060-9-CRISPResso_Report_q0_g4o36.zip!/CRISPResso_on_1060_10/1a.Read_barplot.pdf]

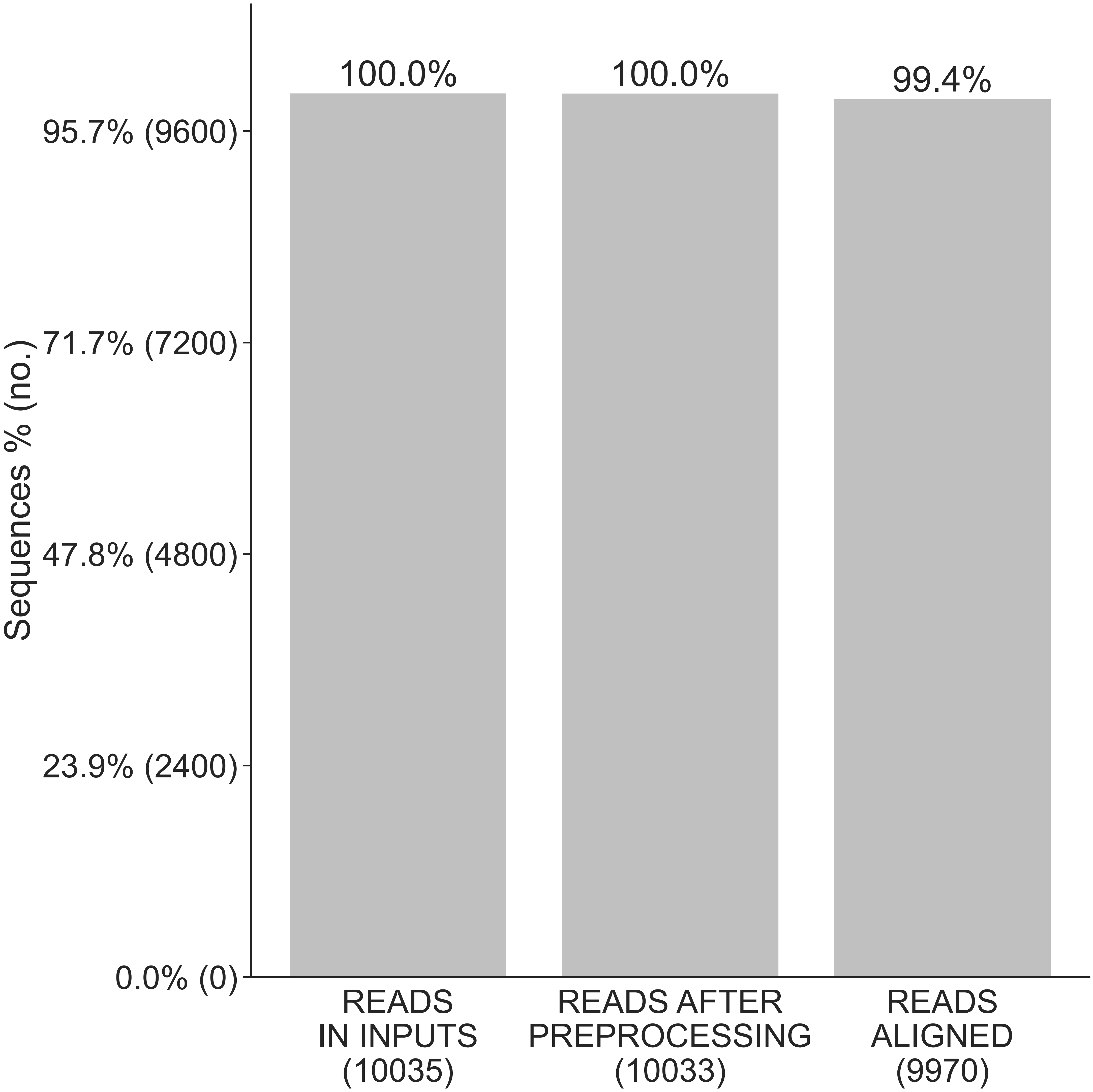

100.0%
100.0%
99.4%
95.7% (9600)
71.7% (7200)
47.8% (4800)
23.9% (2400)
0.0% (0)
Sequences % (no.)
READS
IN INPUTS
(10035)
READS AFTER
PREPROCESSING
(10033)
READS
ALIGNED
(9970)
